# Establishing Sickle Cell Disease Stroke Prevention Teams in Africa is Feasible: Program Evaluation Using the RE-AIM Framework

**DOI:** 10.1097/MPH.0000000000002179

**Published:** 2021-05-18

**Authors:** Djamila L. Ghafuri, Shehu U. Abdullahi, Abdu H. Dambatta, Jamil Galadanci, Musa A. Tabari, Halima Bello-Manga, Nura Idris, Hauwa Inuwa, Aliyu Tijjani, Aisha A. Suleiman, Binta W. Jibir, Safiya Gambo, Awwal I. Gambo, Yusuf Khalifa, Lawal Haliru, Sani Abdulrasheed, Mohammed A. Zakari, Brittany C. Greene, Edwin Trevathan, Lori C. Jordan, Muktar H. Aliyu, Ana A. Baumann, Michael R. DeBaun

**Affiliations:** * Department of Pediatrics, Vanderbilt University Medical Center, Vanderbilt-Meharry Center of Excellence in Sickle Cell Disease; ¶¶ Department of Pediatrics, Division of Pediatric Neurology; ## Health Policy, Vanderbilt Institute for Global Health, Vanderbilt University Medical Center; ∥∥ Department of Neurology, Vanderbilt University Medical Center, Vanderbilt University, Nashville, TN; *** Brown School of Social Work, Washington University of St. Louis, St. Louis, MO; Departments of † Pediatrics; ‡ Radiology; §§ Histopathology, Bayero University/Aminu Kano Teaching Hospital; § Department of Computer Science, Bayero University; Departments of # Radiology; ** Pediatrics, Murtala Mohammed Specialist Hospital; †† Department of Administration, Aminu Kano Teaching Hospital, Kano; Departments of ∥ Radiology; ¶ Hematology and Blood Transfusion; ‡‡ Department of Pediatrics, Barau Dikko Teaching Hospital/Kaduna State University, Kaduna, Nigeria

**Keywords:** sickle cell, stroke prevention, low-resource setting, Nigeria, RE-AIM

## Abstract

We used the *Reach, Effectiveness, Adoption, Implementation*, and *Maintenance* (RE-AIM) framework to evaluate a Stroke Prevention Team’s readiness to prevent strokes in children with sickle cell anemia living in northern Nigeria. The NIH sponsored Stroke Prevention Trial in Nigeria included a goal of a sustainable stroke prevention program. The program’s 1-year *reach* for transcranial Doppler screening was 14.7% (4710/32,000) of which 6.0% (281/4710) had abnormal velocities (≥200 cm/s). All participants with abnormal transcranial Doppler velocities were started on hydroxyurea (*effectiveness*). The leaders of all 5 hospitals agreed to *adopt* the program. After 1 year, program-*implementation* and *maintenance* rates were 100%, demonstrating the program’s feasibility and short-term sustainability.

In the American Society of Hematology (ASH) 2020 guidelines for preventing and treating cerebrovascular disease in sickle cell disease, the committee recommends screening for abnormal transcranial Doppler (TCD) velocities in children with sickle cell anemia (SCA) and treatment with regular blood transfusions for at least 1 year.^1^ After 1 year of regular blood transfusion therapy, hydroxyurea therapy may be used in children without cerebral vasculopathy.^2^,^3^ As a result of standard care practice in high-income countries, the stroke incidence rate has dropped in children with SCA by 10-fold.^4^,^5^ Among children with abnormal TCD velocities, ~20% will have a stroke over 2 years if not treated with regular blood transfusion or hydroxyurea therapy, based on an incidence rate of 10.9 strokes per 100 person-years.^4^ In low-income countries where blood transfusion is costly and not practical, such as Nigeria, the ASH guidelines recommend hydroxyurea therapy as an initial therapy for stroke prevention.^1^

As part of the capacity building component of the phase III Stroke Prevention Trial in Nigeria study (SPRING, Clinicaltrials.Gov: NCT02560935) that tested low-dose (10 mg/kg/day) and moderate-dose (20 mg/kg/day) of hydroxyurea for primary stroke prevention, one aim focused on developing a sustainable program for stroke prevention in SCA.^6^ Thus, we tested the hypothesis that implementing a primary stroke screening program would be a feasible and sustainable strategy for at least a year. We describe the evaluation of the stroke prevention program using the *Reach, Effectiveness, Adoption, Implementation*, and *Maintenance* (RE-AIM) framework.^7^

## MATERIALS AND METHODS

### Study Design, Setting, and Recruitment

A multicenter capacity-building program for establishing *Sickle Cell Disease Stroke Prevention Teams* was conducted from April 2016 to July 2019. Vanderbilt Institutional Review Board and the ethics review committees of participating clinical sites approved this observational study to build capacity. All participants were consented before enrollment. The clinical sites for implementing a regional stroke prevention effort included 5 major hospitals in Kano and Kaduna states, northern Nigeria, Table 1, providing medical care for an estimated 32,000 children with SCA ages 5 to 12.^8^ This total population is an estimate due to the lack of electronic health records and newborn screening programs.

**TABLE 1:** Primary Stroke Prevention Maintenance 12 Months Post-Program Implementation at the 5 Participating Sites in Kano and Kaduna, Nigeria

Study Site	Minimum Estimated Number of Children With SCD Seen Weekly (N)	Estimated Wait Time to be Seen in SCD Clinic (Hr)	TCD Evaluation as Standard of Care 12 Months Post-Implementation on Clinic Days	Children Receiving TCD Services on Clinic Day (N)	Children Receiving TCD Services on Non-Clinic Days (N)
Aminu Kano Teaching Hospital, Kano	80	2	Yes, Tuesday	15	10
Murtala Muhammad Specialist Hospital, Kano	360	2	Yes, Monday-Friday	15	30
Hasiya Bayero Pediatric Hospital, Kano*	108	3	Yes, Wednesday	15	0
Muhammad Abdullahi Wase Specialist Hospital, Kano*	42	2	Yes, Tuesday and Thursday	3-4	5
Barau Dikko Teaching Hospital, Kaduna	45	2	Yes, Thursday	18	10

Aminu Kano Teaching Hospital, Murtala Muhammad Specialist Hospital, Barau Dikko Teaching Hospital are the clinical trial sites for the SPRING trial.

* Hasiya Bayero Pediatric Hospital and Muhammad Abdullahi Wase Specialist Hospital in Kano were the referral sites for the trial. All sites provided TCD screening as standard of care for children with sickle cell anemia ages 5 to 12 years old.

SCA indicates sickle cell anemia; SPRING, Stroke Prevention Trial in Nigeria study; TCD, transcranial Doppler velocity.

### Intervention Program

Each hospital’s leader was initially contacted to assess their capacity to deliver primary stroke prevention strategies for children with SCA. After the assessment was completed, a uniform strategy was decided upon, and the leaders of the 5 collaborating hospitals signed a Memorandum of Understanding after ~4 years of discussions. The core components of the intervention program included the following:

### Identifying Medical Officers and Nurses Willing to Perform TCD Screening

Before starting the trial, the institutional practice was that only radiologists could perform TCD assessment. We initially identified non-radiologists and radiologists to conduct TCD screening and, subsequently, nurses because only 0 to 300 radiologists are available for a population of over 175 million which is approximately one radiologist for 700,000 people.^9^–^11^ There are insufficient radiologists to provide screening TCD evaluations for ~32,000 children with SCA in the region.

### TCD Ultrasonography and Neurological Evaluation Training Program

On the basis of the STOP protocol,^4^ non-imaging TCD training and certification were initiated for the Primary Stroke Prevention in Children with SCA in Nigeria (SPIN) trial at AKTH in Kano, Nigeria.^12^ Two Nigerian radiologists recruited for the trial were trained and certified after demonstrating reproducibility in assessing velocities in the middle cerebral arteries with an experienced TCD ultrasonographer.^12^

For the training, both children with and without SCA were recruited, including those with known abnormal TCD velocities. The TCD-certified non-radiologist physicians and nurses were also trained and certified on the Pediatric NIH Stroke Scale (PedNIHSS), a validated, standardized neurological examination assessing and quantifying the severity of strokes in children.^13^–^15^ An experienced pediatric neurologist traveled to Nigeria (E.T.), and 2 pediatric neurologists in Nashville (F.K. and L.J.) observed the use of the PedNIHSS in physicians (medical officers) and nurses. Each participant performed a minimum of 10 observed neurological examinations in children. The PedNIHSS was used to confirm a normal neurological examination on the same day as TCD and assess children with suspected acute stroke.

### Evaluation: Using the RE-AIM Framework

We selected the RE-AIM framework to evaluate and assess the impact of the *Sickle Cell Disease Stroke Prevention Team* program implementation in northern Nigeria.^16^ The RE-AIM framework includes 5 dimensions: *Reach, Effectiveness, Adoption, Implementation*, and *Maintenance.* In this study, we report the *RE-AIM* factors on a 0% to 100% scale.

#### Reach

The *reach* of the intervention measures the participation rates and representativeness of individuals who participate in the program at each hospital. We defined the numerator of *reach* as the proportion of children with SCA ages between birth to 12 years old that received a TCD screening (primary stroke prevention) or were screened for a stroke (secondary stroke prevention) 1 year after program initiation. We defined the denominator as the number of children with SCA, from birth to 12 years of age, evaluated from April 2016 to July 2019, at the participating hospitals in Kano and Kaduna, Nigeria. We recognize that TCD screening does not start until 2 years of age; however, we included all children followed at each clinic for consistency across each site.

#### Effectiveness

Given that the *Sickle Cell Disease Stroke Prevention Team* program was a research-based program delivered daily by health care providers (physicians and nurses) as standard care in an outpatient clinic setting, *effectiveness* data are reported as part of RE-AIM. Our primary outcome variable was the number of children with abnormal TCD velocities at risk for an initial stroke who initiated hydroxyurea treatment for primary stroke prevention. All children with abnormal TCD velocities were informed about disease-modifying therapy and were offered regular blood transfusion therapy and hydroxyurea therapy for primary stroke prevention.

#### Adoption

*Adoption* includes an assessment of the delivery settings (i.e., intervention locations) and the participation rate of delivery agents involved in implementing the program. We defined *adoption* as the number of certified delivery agents (physicians and nurses) who agreed to adopt and initiate the program.

#### Implementation

*Implementation* was measured by determining whether the intervention was delivered as intended. *Implementation* of the *Sickle Cell Disease Stroke Prevention Program* was analyzed as follows: (1) After program initiation, each institution completed fidelity checks during weekly phone calls between team members in Nigeria and the Data Coordinating Center at Vanderbilt Univrersity Medical Center: (a) number of eligible children seen and receiving TCD screening per week; (b) number of abnormal TCD velocities; (c) the number of children with abnormal TCD velocities referred for hydroxyurea therapy; (d) the number of children receiving hydroxyurea therapy. (2) The participants’ training attendance and TCD certification were recorded and analyzed.

#### Setting-level Maintenance

Setting-level *maintenance* was defined as the extent to which the program became part of routine hospital’s organizational processes and maintained effectiveness. *Maintenance* at 12 months post-implementation was assessed by computing the percentage of participating hospitals that continued participating in the intervention and if the delivery components of the intervention program were still maintained with the same rigor.

## RESULTS

**FIGURE 1:**
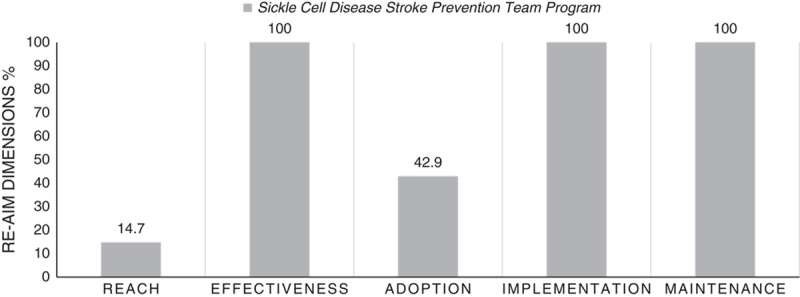
Performance of Sickle Cell Disease Stroke Prevention Team program on individual *Reach, Effectiveness, Adoption, Implementation,* and *Maintenance* (RE-AIM) dimensions, combined from 5 hospitals in Kano and 1 hospital in Kaduna, Nigeria. SCA indicates sickle cell anemia.

**FIGURE 2:**
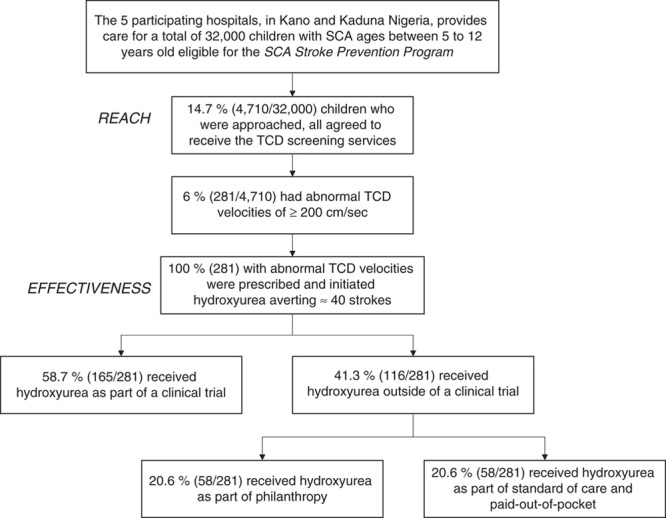
Flow diagram describing: (1) *Program Reach* defined as the proportion of children followed in the sickle cell disease clinics at the 5 hospitals receiving transcranial Doppler (TCD) screening as standard care; and (2) *Program Effectiveness*, defined as the number of children with abnormal TCD velocities at risk for an initial stroke who initiated hydroxyurea treatment as a form of primary stroke prevention, at the 4 hospitals in Kano and 1 hospital in Kaduna, Nigeria. SCA indicates sickle cell anemia.

Before our program intervention, no systematic approach for primary stroke prevention was utilized in the region. As result of the program, children between 2 and 12 years of age received TCD screening free-of-charge as standard care in 5 hospitals. Hydroxyurea therapy was provided free-of-charge for children with abnormal TCD velocities. Figure 1 shows the *RE-AIM* factors on a 0% to 100% scale and their application in the evaluation of the *Sickle Cell Disease Stroke Prevention Team* program. Figure 2 is a flow diagram depicting the study *reach* and *effectiveness*.

### Reach

Hospital leaders at the 5 eligible hospitals with established pediatric SCD clinics in metropolitan Kano and Kaduna, northern Nigeria, agreed to participate in the-*Sickle Cell Disease Stroke Stroke Prevention Team* program. From April 2016 to July 2019, ~32,000 children attending the SCD clinics were identified and found to be eligible for the Stroke Prevention program. All children with SCA between 2 and 12 years of age, who visited the participating hospitals and were approached, agreed to receive the TCD evaluation, Table 2. The program *reach* was 14.7%, that is, 4710 of ~32,000 children with SCA received the TCD screening services. Of those children receiving TCD screening, 6% (281/4710) had confirmed abnormal TCD velocities.

**TABLE 2:** Baseline Demographics and Characteristics of Children With Sickle Cell Anemia Ages Between 2 to 12 Years Receiving TCD Screening as Standard Care at the Our Hospitals in Kano and 1 Hospital in Kaduna, Nigeria

Variables	Children With SCA* (N=3296)
Age, median (IQR) (y)	7.0 (6)
Sex, male (%)	50.8
TAMMV, mean (SD) (cm/s)	140.13 (32.28)
Normal (<170 cm/s) (%)	79.7
Conditional (170-199 cm/s) (%)	12.0
Abnormal (≥200 cm/s) (%)	6.1
No signal (%)	1.9

* Children with SCA living in Kano Nigeria, who were approached and agreed to receive the TCD screening with complete data on age and sex.

IQR indicates interquartile range; TAMMV TCD, time-averaged mean of the maximum velocity of transcranial Doppler value; SCA, sickle cell anemia.

Children with pre-existing strokes, not eligible for primary stroke prevention but eligible for secondary stroke prevention, were evaluated at 4, not 5 hospitals because the fifth hospital’s clinical staff was not set up to address the presence of pre-existing strokes. Among the children with SCA assessed for pre-existing strokes, 5.6% (172/3049) of the children were identified as having an affirmative answer to the 10 screening stroke questions,^14^,^15^ a validated questionnaire for detecting moderate to severe neurological impairment in children living in Africa, and had a subsequent history and physical examination that confirmed they had a stroke.^14^,^15^ Furthermore, only 4 of the 5 participating hospitals asked the 10 stroke screening questions at the beginning of each clinic visit. Due to limited clinic space, the questions were asked in a small group format, often with 10 to 15 guardians with at least as many children in a group.

### Effectiveness

Regarding the *effectiveness* outcome (primary stroke prevention), before the program implementation, none of the children were prescribed moderate-fixed dose (20 mg/kg/day) hydroxyurea therapy for primary stroke prevention. After the program implementation, all physicians prescribed hydroxyurea either low (10 mg/kg/day) or moderate fixed-dose (20mg/kg/day) based on chaired decision making with the guardians for children with abnormal TCD velocities. All guardians of children with confirmed abnormal TCD velocities rejected the initial offer of blood transfusion therapy for their children.

All children with abnormal TCD velocities received hydroxyurea manufactured by Bond Chemical Industries, Nigeria. Of the 281 children with confirmed abnormal TCD velocities, 20.6% (58/281) received hydroxyurea free-of-charge from philanthropic donations, 20.6% paid out-of-pocket, and the remaining children received hydroxyurea as part of the clinical trial (58.7%; SPRING trial). To address the inability of many families to pay out-of-pocket for hydroxyurea, recently, the Director-General of Hospital Management Board in Kano, Nigeria, and the Minister of Health in Kaduna, Nigeria, agreed to provide hydroxyurea free-of-charge to all children with SCA and abnormal TCD velocities.^8^ In the current cohort, treating 281 children with SCA with abnormal TCD velocities presumably averted ~40 strokes using the number-needed-to-treat to prevent 1 stroke (NNT=7) from the STOP trial and assuming equal efficacy, ~<1 event per 100 person-years.^4^,^17^

For the *effectiveness* of secondary stroke prevention, prescribing disease-modifying therapy (hydroxyurea) was also high (100%). No guardian elected for transfusion therapy outside of the clinical trial setting; 98.8% (170/172) of participants identified as having a stroke received hydroxyurea therapy for secondary stroke prevention.

### Adoption

All 5 hospitals providing care for children with SCD in Kano and Kaduna in northern Nigeria were involved in the adoption. For primary stroke prevention, adoption at the provider level included the following: of the eligible 28 physicians (20 non-radiology physicians and 8 radiologists) at the participating sites, 60.7% (17/28) were approached and agreed to TCD ultrasonography training for primary stroke prevention; 42.9% (12/28) received certification.

### Implementation

Overall, at 12-month evaluation, 100% of the originally planned components of the program for primary stroke prevention for children with SCA were implemented at all 5 sites. The following objectives were achieved: (1) all 5 hospital leaders created a *Sickle Cell Disease Stroke Prevention Team*, and 42.9% (12/28) of radiology and non-radiology physicians were successfully certified in TCD ultrasonography; (2) all sites and delivery agents agreed to provide screening services in their outpatient SCD clinics.

### Maintenance

At 12 months post-initiation of the program, all the initially planned program components were implemented per protocol, including hands-on, one-on-one TCD training and TCD certification, PedNIHSS training, and certification of physicians and nurses. All stroke teams continued to maintain and participate in the program, financially supporting a certified TCD ultrasonographer, a certified nurse in standardized neurological examination, and routine screening of children with SCA, with TCD evaluation during outpatient clinic visits, Table 1.

Seven health care workers (2 nurses and 5 physicians) at the 5 hospitals agreed to adopt the program. After 4 years of interaction, the Director-General of Hospital Boards in Kano and Kaduna states agreed to sign a Memorandum of Understanding to maintain program sustainability. In both states, a gap of ~3 and 6 months, respectively, occurred between activation of the Memorandum of Understanding when the state leaders agreed to provide the hydroxyurea free-of-charge for children at high-risk of an initial or subsequent stroke. Before the state provided hydroxyurea free-of-charge, the team members paid for the hydroxyurea out of pocket, and a health care administrator secured additional funds. The children receiving hydroxyurea are being monitored as part of the new standard of care by the multidisciplinary *Sickle Cell Disease Stroke Prevention Teams* at each site bimonthly, with complete blood count evaluation every 6 months. There is no hydroxyurea dose escalation based on the results of the SPIN Trial, showing adequate stroke prevention with moderate fixed-dose hydroxyurea (20 mg/kg/d).^12^

## DISCUSSION

We used the RE-AIM framework to evaluate the impact of implementing the *Sickle Cell Disease Stroke Prevention Teams* program over 1 year in Kano and Kaduna states, Nigeria, to identify “why” and “how” this intervention works in this setting. Creating *Sickle Cell Disease Stroke Prevention Teams*, including a physician, a skilled TCD ultrasonographer, and a skilled nurse, all trained in performing appropriate neurological examinations, will allow for additional children to be reached. Understanding the barriers and facilitators of program adoption can inform its generalizability to other settings and scalability to other populations.^18^–^20^ To our knowledge, our *Sickle Cell Disease Stroke Prevention Team* program for children with SCA is one of the first intervention programs for a region in Africa, not an individual hospital. This program is feasible in northern Nigeria and may be generalizable to other areas of Africa.

Before our intervention, primary stroke prevention strategies were not embedded as the standard care for children with SCA in northern Nigeria. All 5 hospital leaders agreed to adopt the program strategies, and all components were implemented and maintained for at least 12 months postimplementation, as planned, Table 1. Reach was limited by the high volume of children with SCA, lack of trained personnel certified to perform TCD, and the paucity of TCD equipment. The training of the physicians and nurses was well-received. The program gained support and acceptability among the local leaders and health professionals before the signed Memorandum of Understanding. To increasee the reach a total of TCD machines were purchased with philanthropy funds and all TCD examinations were free of charge.

All guardians of children with abnormal TCD velocities or pre-existing strokes refused initial regular blood transfusion therapy. Reasons guardians did not accept monthly blood transfusion therapy for primary and secondary stroke prevention include, but are not limited to, the regional practice of requiring families to regularly seek blood donors to replace the units used and the high cost of red blood cell unit relative to income. The mean annual cost of chronic transfusion (without chelation) is $3276 United States dollars.^21^ Approximately 40% of the Nigerian population (~83 million people) live below Nigeria’s poverty line, ∼US$1.00.^22^ The large discrepancy between the cost to prevent strokes and the annual income is a significant barrier to blood transfusion therapy for primary stroke prevention. Our experience is identical to that of Lagunju et al at a single site in Nigeria where 100% of families refused regular blood transfusion therapy for primary and secondary stroke prevention.^21^ Some Nigerians can afford monthly blood transfusion therapy and iron chelation, but most cannot.

Significant barriers for SCA stroke prevention included the lack of TCD machines in the region, the potential charge for performing TCD examination, the scarcity of TCD-certified sonographers, the slow development of state hospital leaders, and partnerships to activate the Memorandum of Understanding. In addition, the cost of imported hydroxyurea is prohibitively for most families in the region. *Sickle Cell Disease Stroke Prevention Teams* are feasible in northern Nigeria and elsewhere in Africa, with meaningful partnerships between regional public health care administrators, health care providers, state government leaders, and local philanthropists. Together these entities can ensure the sustainability of stroke prevention programs for children with SCA.

We have demonstrated the feasibility of implementing *Sickle Cell Disease Stroke Prevention Teams* in northern Nigeria. The cornerstone of the Stroke Prevention Team’s success was the guiding philosophy that Nigerians should provide sustainable stroke prevention services for Nigerian children with SCA. This philosophy led to 2 critical efforts that strengthened the team’s resolve. First, finding a Nigerian pharmaceutical company (Bond Chemical Industries, Nigeria) that produces hydroxyurea at a subsidized cost (US$0.16 for 500 mg capsule per day). Second, the Nigerian team’s steady incremental belief that they could make a difference in children’s lives with SCA. The team’s raison d’etre was manifested in their enthusiasm to obtain TCD and stroke detection training, coupled with the team’s willingness to pay-out-of-pocket for hydroxyurea in children with abnormal TCD velocities before the state-supported free hydroxyurea therapy was available.
